# Snake kolmiovirus encodes a single form of delta antigen and shows no evidence of translation from ORF2

**DOI:** 10.1099/jgv.0.002327

**Published:** 2026-10-07

**Authors:** Leonóra Szirovicza, Udo Hetzel, Tomas Strandin, Anja Kipar, Jussi Hepojoki

**Affiliations:** 1 Medicum, Department of Virology, University of Helsinki, Helsinki, Finland; 2 Institute of Veterinary Pathology, Vetsuisse Faculty, University of Zürich, Zürich, Switzerland; 3 Department of Veterinary Biosciences, Faculty of Veterinary Medicine, University of Helsinki, Helsinki, Finland

**Keywords:** deltavirus, delta antigen, hepatitis D virus, kolmiovirus, satellite virus

## Abstract

Hepatitis D virus (HDV) is a satellite virus that utilizes hepatitis B virus (HBV) as a helper for infectious particle formation. HDV was originally identified as a novel antigen in liver biopsies of HBV patients, and later studies showed the ‘delta’ antigen (DAg) to be the sole protein encoded by HDV. Until the discovery of HDV-*like* agents in birds and snakes in 2018, HDV was a unique example of animal satellite viruses. We identified Swiss snake colony virus 1 (SwSCV-1) in the brain of a *Boa constrictor* and through comparison we found the genome organization of SwSCV-1 to resemble that of HDV. However, in addition to the DAg ORF, the genome of SwSCV-1 includes another >500 nt ORF, ‘ORF2’. To study whether the putative ORF2-encoded protein plays a role in the SwSCV-1 life cycle, we established an infectious clone of the virus with a point mutation in the methionine initiation codon of ORF2. The mutation did not significantly affect initiation of replication, establishment of persistent infection or infectious particle formation upon superinfection with a helper virus. Using additional methods, we gathered further evidence confirming that ORF2 is not actively translated in boa constrictor cells. We further showed that unlike HDV, SwSCV-1 expresses a single form of the DAg. Although the proteins encoded by SwSCV-1 and HDV only include one and two forms of the DAg, respectively, whether other kolmioviruses express additional forms of DAg or related proteins in some cell types or host species merits further research.

**Impact Statement**

Satellite viruses with similar genome organization were found in various animals ~40 years after the discovery of hepatitis D virus (HDV), thereby giving rise to the family Kolmioviridae. HDV encodes a single protein, the delta antigen (DAg), which comes in small and ~20 amino acids longer large form. The genome of some HDV species and many of the newly found kolmioviruses contains additional ORFs, potentially enabling protein expression. Here, we studied the viral proteins expressed during Swiss snake colony virus 1 (SwSCV-1) infection of boa constrictor cells. Our findings show that unlike HDV, SwSCV-1 encodes only a single form of DAg. In addition, our study suggests that, like in HDV, the additional ORF in SwSCV-1 genome does not give rise to a protein. Although we could not demonstrate expression of additional viral proteins during SwSCV-1 infection, it is important to study the proteome of other kolmioviruses.

## Introduction

Hepatitis D virus (HDV) – first described in 1977 – is a unique pathogen, originally identified in liver biopsies of hepatitis B virus (HBV) carriers [1]. Soon after its discovery, chimpanzee experiments allowed the researchers to recognize HDV as a satellite virus of HBV, needing the glycoproteins of the latter for infectious particle formation [2, 3]. HDV has a negative-sense circular ssRNA genome of ~1.7 kb, which – similarly to viroids – replicates via a double rolling circle mechanism [4, 5]. Three different HDV RNA species are present in the cell during its replication cycle: the genome and the antigenome, which are exact complements of each other, and the ~800 nts long mRNA, which gives rise to the delta antigen (DAg) [5]. During HDV replication, multimeric RNA species are generated; these are processed into unit-length monomers by ribozymes that are present in both the genomic and antigenomic RNAs [6]. In case of HDV, the DAg exists in two forms [small (S-DAg) and large (L-DAg)], due to the RNA editing activity of the cellular enzyme adenosine deaminase acting on RNA (ADAR) [7–9]. As the consequence of the ADAR editing on the antigenomic HDV RNA strand [10], the stop codon of the DAg ORF is modified into a tryptophan codon, resulting in elongation of the DAg by 19 additional amino acids to generate the L-DAg [11]. The S-DAg promotes replication, while the L-DAg has an inhibitory effect on the replication and shifts the cycle towards particle formation [12]. Post-translational modifications such as phosphorylation, acetylation and isoprenylation likely contribute to the vastly different roles that the two forms of the DAg play during HDV replication [12, 13].

For more than 40 years, HDV was the sole representative of the unassigned genus *Deltavirus* [14]. In 2018, coincidentally and independently of Wille and colleagues, who identified a ‘novel’ HDV-*like* agent in ducks [15], we identified an HDV-*like* agent in the brain of a *Boa constrictor* [16]. In both cases, the HDV-*like* virus appeared to exist without a co-infecting hepadnavirus, and in fact, we later demonstrated reptarena- and hartmaniviruses to serve as the helper viruses for the ‘snake deltavirus’, i.e. Swiss snake colony virus 1 (SwSCV-1) [17]. Subsequent reports from different groups described additional HDV-*like* agents in termites, fish, frogs, newts, rodents, bats, deer, woodchucks, birds and even marsupials [18–23], which provoked the International Committee on Taxonomy of Viruses to establish the realm *Ribozyviria* and the family *Kolmioviridae* that currently includes 11 genera [24–26].

While the characterization of the newly identified kolmiovirids is in its infancy, it appears likely that these agents share many of HDV’s characteristics. The HDV genome contains several ORFs; however, the evidence suggests that the two forms of the DAg are the only proteins translated [5, 27]. In addition to the DAg ORF, the genome of SwSCV-1 contains a second ORF (ORF2) in the genomic orientation that would give rise to a putative protein of 177 amino acids [16]. Although not studied for most of the novel kolmioviruses [15, 18–21], a recent study found transcriptomics-based evidence of the presence of mRNA from a genomic-orientation ORF in the *Serinus canaria*-associated deltavirus genome [28]. The researchers did not identify significant homologies to any known protein for the *S. canaria*-associated deltavirus ‘ORF2’ product [28], similar to the SwSCV-1 ORF2 protein product [16].

In this study, we investigated whether the SwSCV-1 ORF2 and its putative protein product play a role in the life cycle of the virus. We first introduced a mutation to remove the ORF2 methionine initiation codon while retaining the intact DAg ORF in the plasmid-driven SwSCV-1 infectious clone. We then compared replication initiation, infectious particle formation and persistence of infection between the ORF2-deleted and wild-type (2× and 1.2×SwSCV-1) genome clones in snake cells. Next, we utilized recombinant protein expression to study the cellular localization of the putative ORF2-encoded protein, and to demonstrate that only a single form of the DAg is produced in SwSCV-1 infected boid cells. Finally, to determine the presence or absence of ORF2 mRNA, we performed directional RNA sequencing utilizing two approaches (rRNA depletion and poly(A) enrichment) for library preparation from RNA extracted from SwSCV-1-infected cells.

## Methods

### Plasmids and cloning

The pCAGGS plasmid-based infectious clones bearing either 2× or 1.2×SwSCV-1 genome are described in [17, 29]. We ordered synthetic genes with 1.2× genome copies of SwSCV-1 (accession number: NC_040729.1), with the methionine initiation codon of the ORF2 mutated to isoleucine (ATG→ATA) leaving the DAg’s amino acid sequence unaffected but changing a serine codon from AGC to AGT (Fig. 1a). In addition, we ordered synthetic genes representing the putative protein translated from ORF2 with and without a stop codon. The absence of the stop codon allows attachment of an HA-tag (encoded in the vector) to the translated protein (Fig. 1b) and subsequent detection with the tag-specific antibody. In addition, we ordered three synthetic genes for the DAg ORF: one with the ORF extending only to the S-DAg stop codon, one with the natural ORF until the end of the putative L-DAg stop codon and one with the genome-encoded stop codon altered to a tryptophan codon (TAG→TGG) to force L-DAg expression (Fig. 1c). All synthetic genes were made by GeneUniversal. We subcloned the inserts into pCAGGS-MCS [30] following the protocol described in [17]. In brief, after FastDigest (Thermo Fisher Scientific) restriction digestion with suitable enzymes and agarose gel electrophoresis of the inserts, the GeneJET Gel extraction kit (Thermo Fisher Scientific) served to purify the products. After linearization and blunting, in the case of the infectious clones, of the pCAGGS/MCS [30] plasmid, T4 DNA ligase (Thermo Fisher Scientific) was used to ligate the plasmid and the inserts. We used the ligation products to transform chemically competent *Escherichia coli* (DH5α strain) and subsequently plated the cells on Luria broth (LB) agar plates with 100 µg ml^−1^ of ampicillin and incubated overnight (O/N) at 37 °C. We picked and transferred single colonies into 5 ml of LB medium (10 g l^−1^ tryptone, 10 g l^−1^ NaCl, 5 g l^−1^ yeast extract), incubated them O/N at 37 °C at 220 r.p.m., and isolated plasmids using the GeneJET Plasmid Miniprep Kit (Thermo Fisher Scientific) from 2 ml of the O/N culture. Sanger sequencing (provided by the DNA Sequencing and Genomic Laboratory, Institute of Biotechnology, University of Helsinki) confirmed the presence of the correct inserts. ZymoPURE II Plasmid Maxiprep Kit (Zymo Research) following the manufacturer’s protocol served for preparing endotoxin-free plasmid stocks.

**Fig. 1.**
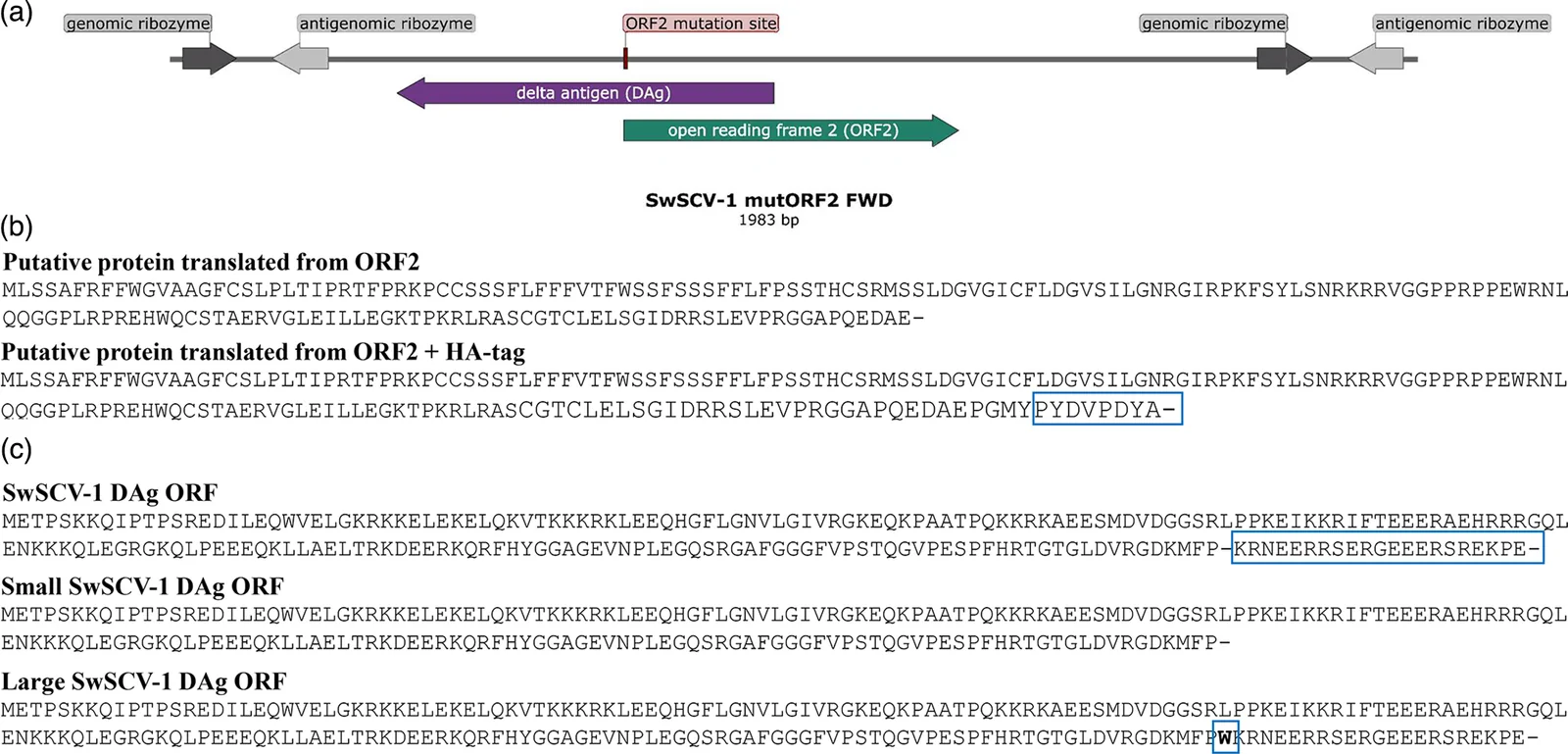
Schematic representation of the synthetic genes and protein products. (**a**) Schematic representation of the 1.2× genome SwSCV-1 insert in forward (FWD)/genomic orientation with the introduced point mutation in the ORF2. The point mutation (ATG→ATA) in the ORF2 initiation codon inhibits translation of the putative protein, while leaving the DAg unaffected. (**b**) Amino acid sequence of the ORF2 protein translated with or without an HA-tag (marked with a blue rectangle) from the expression construct. (**c**) Amino acid sequence of the proteins translated from the synthetic genes representing the three forms of the SwSCV-1 DAg: the full unedited version, allowing RNA editing (site marked by the blue rectangle); the small form without the possibility of editing and extension; and the edited DAg (editing site marked by the blue square), forcing the expression of the large form of the protein.

### Cell culture and superinfection

The study made use of a *B. constrictor* kidney cell line, I/1Ki, spontaneously immortalized from kidney homogenate [31], and two persistently SwSCV-1-infected cell lines, I/1Ki-∆ and I/1Ki-1.2×∆ [17, 29]. To generate the I/1Ki-mutORF2Δ cell line, we transfected I/1Ki cells with the 1.2×SwSCV-1 genome clone with disabled ORF2, followed by continuous passaging and maintenance of the cells. In addition to the above-mentioned cell lines, we utilized four persistently SwSCV-1-infected cell lines (V/5Lu-Δ, V/1Ki-Δ, V/2Hz-Δ and V/5Liv-Δ) described in [17]. One further persistently SwSCV-1-infected cell line was established, designated V/4Br-Δ. Briefly, we transfected previously described [32] boa constrictor brain cells (V/4Br) with the 1.2×SwSCV-1 forward (FWD) infectious clone [29], followed by continuous passaging of the cells as described for the above-mentioned Δ cell lines [17].

In addition to the snake cell lines, we used human hepatocellular carcinoma (Huh7) and human embryonic kidney (HEK293T) cells. Both cell lines were obtained from ATCC.

We maintained all snake cell lines in Minimal Essential Medium Eagle (Sigma Aldrich), Huh7 cells in Dulbecco’s modified Eagle’s medium (low glucose) (Sigma Aldrich) and HEK293T cells in Dulbecco’s modified Eagle’s medium (high glucose) (Sigma Aldrich). All media were supplemented with 10% FBS (Gibco), 200 mM l-glutamine (Sigma Aldrich), 100 µg ml^−1^ of streptomycin (Sigma Aldrich) and 100 U ml^−1^ of penicillin (Sigma Aldrich) in an incubator at 30 °C with 5% CO_2_.

For infectious particle formation studies of the persistently SwSCV-1-infected cell lines, we inoculated the cells with Haartman Institute snake virus (HISV-1) [33], shown to efficiently function as the helper virus of SwSCV-1 [17, 29]. We used 600 copies of HISV-1 S segment RNA per cell as the infectious dose, which corresponds to ~3 m.o.i. [34].

### Transfection

Lipofectamine 2000 (Thermo Fisher Scientific) served as the reagent to transfect I/1Ki cells, as described [17, 35, 36]. For Huh7 and HEK293T cells, the Fugene HD (Promega) reagent served for transfection purposes, as described in [36]. Briefly, we prepared mixtures of 500 ng plasmid DNA in 50 µl OptiMEM (Thermo Fisher Scientific) and 3 µl of Lipofectamine 2000 in 47 µl of OptiMEM (Thermo Fisher Scientific). Then, we combined the mixtures by pipetting up and down and allowed complex formation for 15–30 min at room temperature (RT). Fugene HD was added directly into the DNA-OptiMEM mixture before allowing complex formation. We mixed 1 ml trypsinized cell suspension (~1.8 cm^2^ of cells per ml, i.e. a confluent 75-cm^2^ suspended into 38 ml of fully supplemented medium) into the transfection reagent/plasmid mixture and allowed the suspension to stand at RT for 15–30 min before plating, and at 5–6 h post-plating, we replaced the transfection mixture with fully supplemented growth medium. Depending on the upcoming experiment, we scaled up the above reaction volumes accordingly.

### Western blot

For Western blot (WB), we scraped the cells into PBS, pelleted them by centrifugation (500 ***g***, 5 min), resuspended the pellets in RIPA buffer [50 mM Tris, 150 mM NaCl, 1% Triton-x-100, 0.1% SDS, 0.5% sodium deoxycholate, protease inhibitor cocktail (Roche)] and measured the protein concentration of the samples using the Pierce™^T^ BCA Protein Assay Kit (Thermo Fisher Scientific). We separated an equal amount of protein for each sample on SDS-PAGE using 4–20% Mini-PROTEAN^®^ TGX gels (Bio-Rad), transferred onto nitrocellulose and immunoblotted as described [37]. We used rabbit α-SwSCV-1 DAg antiserum [16] as the primary antibody at 1 : 2,000 dilution. For detection of proteins containing an HA-tag, a mouse anti-HA (clone 16B12; BioLegend) primary antibody was used at 1 : 1,000 dilution. In addition, the serum of the boa constrictor, from which we originally identified SwSCV-1 [16], was also utilized for staining at 1 : 250 dilution, visualized by anti-IgY boa constrictor antibody [37] labelled with Alexa Fluor 680 NHS ester (Thermo Fisher Scientific) following the manufacturer’s protocol. The other secondary antibodies used were as follows: IRDye 680RD donkey anti-mouse (IgG) (LI-COR Biosciences) and IRDye 800CW donkey anti-rabbit (IgG) (LI-COR Biosciences), both at 1 : 10,000 dilution. Lab Vision™^T^ pan-actin mouse monoclonal antibody (Thermo Fisher Scientific) at 1 : 200 dilution served to detect β-actin as a loading control, and the Odyssey Infrared Imaging System (LI-COR Biosciences) for recording the results.

### Immunofluorescence staining

For immunofluorescence (IF) staining of cells, we followed a described protocol [17]. Briefly, we plated the cells on collagen-coated CellCarrier-96 Ultra plates (PerkinElmer), fixed them with 4% paraformaldehyde (PFA) in PBS for ~15 min at RT, incubated 15 min at RT in permeabilization buffer (50 mM Tris, 150 mM NaCl, 0.25% Triton-X-100, pH 7.4) supplemented with 3% BSA to reduce unspecific binding and to quench the remaining PFA and directly proceeded with staining. We used the directly labelled anti-SDAg-AF488 antibody described in [17] at 1 : 500 dilution or rabbit anti-SwSCV-1 DAg antiserum at 1 : 1,000 dilution. Alexa Fluor 488- or 647-labelled donkey anti-rabbit or anti-mouse immunoglobulin (Thermo Fisher Scientific) at 1 : 1,000 dilution served as the secondary antibodies. For the detection of proteins with an HA-tag, we used the mouse anti-HA (clone 16B12) primary antibody at 1 : 400 dilution. For imaging of the plates stored in the dark at 4 °C, we used the Opera Phenix High Content Screening System (PerkinElmer), provided by FIMM (Institute for Molecular Medicine Finland) High Content Imaging and Analysis (FIMM-HCA).

### Quantitative reverse transcription PCR

Quantitative reverse transcription PCR (qRT-PCR), described in [29], served for quantification of SwSCV-1 RNA in cells. RNAs isolated using the GeneJET RNA Purification Kit (Thermo Fisher Scientific) were used as the template for 10 µl reactions set up according to the TaqMan^®^ Fast Virus 1-Step Master Mix (Thermo Fisher Scientific) protocol. The primers and probes used targeted the genomic strand of SwSCV-1. We ran the samples in duplicate using the AriaMX real-time PCR system (Agilent) with the following cycling conditions: reverse transcription for 5 min at 50 °C; initial denaturation for 20 s at 95 °C; and two amplification steps at 95 °C for 3 s and 60 °C for 30 s, repeated for 40 cycles. The TranscriptAid T7 High Yield Transcription Kit (Thermo Fisher Scientific) was used to generate *in vitro* transcribed SwSCV-1-specific control RNA, which allowed us to generate a standard curve and estimate the number of RNA copies in the samples [29]. In addition, before assembling the qRT-PCR reactions, we subjected the samples to DNase I treatment (Thermo Fisher Scientific) according to the manufacturer’s recommendations to eliminate any transfection leftover plasmid DNA in the RNA preparations.

We normalized the SwSCV-1 RNA levels against a housekeeping gene, using the following primers and probe for the detection of *B. constrictor* phosphoglycerate kinase 1 (PGK-1): forward primer: 5′-GATGGATAAAGTTGTGGAA-3′, reverse primer: 5′-TTCGGTATTCCATTTAGC-3′ and probe 5′ 6-Fam (carboxyfluorescein)-TAGCAGTATCTCCGCCACCAAT-BHQ-1 3′. We used *Python bivitattus* PGK-1 (GenBank accession: XM_007429612.3) as the template in Unipro UGENE [38] for reference assembly to obtain *B. constrictor* PGK-1 mRNA from reads of our earlier metatranscriptomic studies [33, 39–41].

### Near-infrared fluorescent Northern blot

Near-infrared Northern blot analysis, performed as described in [29], served to detect viral RNA in the permanently SwSCV-1-infected cells. Briefly, we isolated RNA from I/1Ki, I/1Ki-2×Δ, I/1Ki-1.2×Δ and I/1Ki-mutORF2Δ cells by TRIzol™ reagent (Thermo Fisher Scientific), and separated the RNA samples in parallel with ssRNA ladder (New England Biolabs) on agarose-formaldehyde gels in tricine/triethanolamine buffer, as described by Mansour and Pestov [42]. Following, we transferred the RNAs from the gel to Hybond™-N^+^ nylon membrane (GE Healthcare) using O/N capillary transfer, then cross-linked the RNA to the membrane by an ultraviolet cross-linker (120 mJ cm^−2^ at 254 nm) (Analytik Jena). After, we prehybridized the membranes to block non-specific binding sites and performed O/N incubation with the fluorescent probes that were diluted into the pre-hybridization buffer to a final concentration of 1 nM. We used probes targeting the ladder, the SwSCV-1 genome and the DAg mRNA as described [29]. The next day, we washed the membranes three times before recording the results utilizing the Odyssey Infrared Imaging System (LI-COR Biosciences).

### Next-generation sequencing, strand-specific RNAseq and Sanger sequencing of SwSCV-1 from infected cells

To investigate the SwSCV-1 genome within the infected cell, we performed metatranscriptomic analysis of the persistently SwSCV-1-infected cells. The NEBNext rRNA depletion kit (New England BioLabs) served for removal of ribosomal RNA from the total RNA isolated from cell pellets using the TRIzol™ Reagent (Thermo Fisher Scientific) according to the manufacturer’s recommendation. The NEBNext Ultra RNA library preparation kit (New England BioLabs) served for next-generation sequencing (NGS) library preparation and indexing of the samples, and the NEBNext Library Quant kit for Illumina (New England BioLabs) allowed quantification of the libraries before sequencing on the Illumina MiSeq sequencer (Illumina) using the MiSeq reagent kit (version 3; Illumina). Lazypipe [43] served for *de novo* assembly of SwSCV-1 genome from the samples. In parallel, reference assembly using Bowtie2 [44] run in Unipro UGENE [38] served to obtain consensus sequences for the SwSCV-1 from different cell lines.

To confirm the presence of the mutation in ORF2, we isolated RNA from I/1Ki-mutORF2Δ cells at ~2 months post-transfection, and synthesized cDNA using the RevertAid First Strand cDNA Synthesis Kit (Thermo Fisher Scientific) according to the manufacturer’s recommendation for random hexamers. The following two primer pairs: FWD-1 5′-CTTTCCGGTACCCCTTGAGT-3′, REV-1 5′-GAACAATGGGTCGAGCTTGG-3′, FWD-2 5′-CTTCCCCAGCTCCTCCATAG-3′, REV-2 5′-AGATCCCTACTCCGTCCAGA-3′ and the Phusion Flash High-Fidelity PCR Master Mix (Thermo Fisher Scientific) used according to the manufacturer’s recommendation served to amplify the region containing the introduced mutation. Sanger sequencing (performed by the DNA Sequencing and Genomic Laboratory, Institute of Biotechnology, University of Helsinki) served to confirm the presence of the ORF2 mutation.

For the strand-specific RNA sequencing, we isolated RNA from a 75 cm^2^ cell culture flask of 1.2×SwSCV-1 FWD-transfected cells (4 weeks post-transfection) using TRIzol reagent (Invitrogen) according to the manufacturer’s recommendation. We split the extracted RNA into two aliquots, which we sent to Azenta Life Sciences for sequencing. Both poly(A) enrichment and rRNA depletion were utilized for RNA selection prior to library preparation. We then aligned to the SwSCV-1 genome and antigenome the reads representing the direction of the original RNA template using Bowtie2 in Ugene.

## Results

### Mutation of the ORF2 does not inhibit initiation of replication

To investigate the potential role of the putative protein encoded by SwSCV-1 ORF2, we established an infectious clone with a single point mutation turning the methionine initiation codon into isoleucine codon while maintaining the amino acid sequence of the DAg. The insert, designed according to the previously described 1.2× genome copy of SwSCV-1 approach [29] is schematically depicted in Fig. 1a. As described for 1.2× and 2× genome SwSCV-1 infectious clones in earlier studies [17, 29], we generated constructs with the insert in both genomic (mutORF2 SwSCV-1 FWD) and antigenomic (mutORF2 SwSCV-1 REV) orientation. Based on our earlier studies, transfection of the FWD construct to the boa constrictor kidney cell line, I/1Ki, leads to DAg expression only in the case of virus replication, while the REV construct will express the protein also through the CAG promoter of the plasmid [17, 29]. To test the ability of the mutORF2 clones to initiate SwSCV-1 replication, we transfected I/1Ki cells with both constructs, fixed or collected cells 1–4 days post-transfection (dpt) and performed IF staining and WB (Fig. 2a and b). Both approaches displayed increasing SwSCV-1 DAg expression over the course of the 4 days for both constructs. WB comparison to the 1.2×SwSCV-1 FWD construct showed comparable migration patterns and increasing DAg expression (Fig. 2c), similarly to our previous observations [29]. To further compare the mutORF2 constructs to the wild-type constructs, we transfected I/1Ki cells with all six clones (2×, 1.2× and mutORF2 SwSCV-1 FWD and REV) and collected cell pellets at 1, 2, 3 and 7 dpt. We quantified the amount of SwSCV-1 RNA at each time point by qRT-PCR after DNAse I treatment to remove potential plasmid DNA carryover. Fig. 2d shows an increasing amount of SwSCV-1 RNA in the transfected cells, indicating replication initiation from all of the constructs at roughly equal efficiency, yielding similar SwSCV-1 RNA levels by day 7. Each time point represents two biological replicates with two technical replicates.

**Fig. 2.**
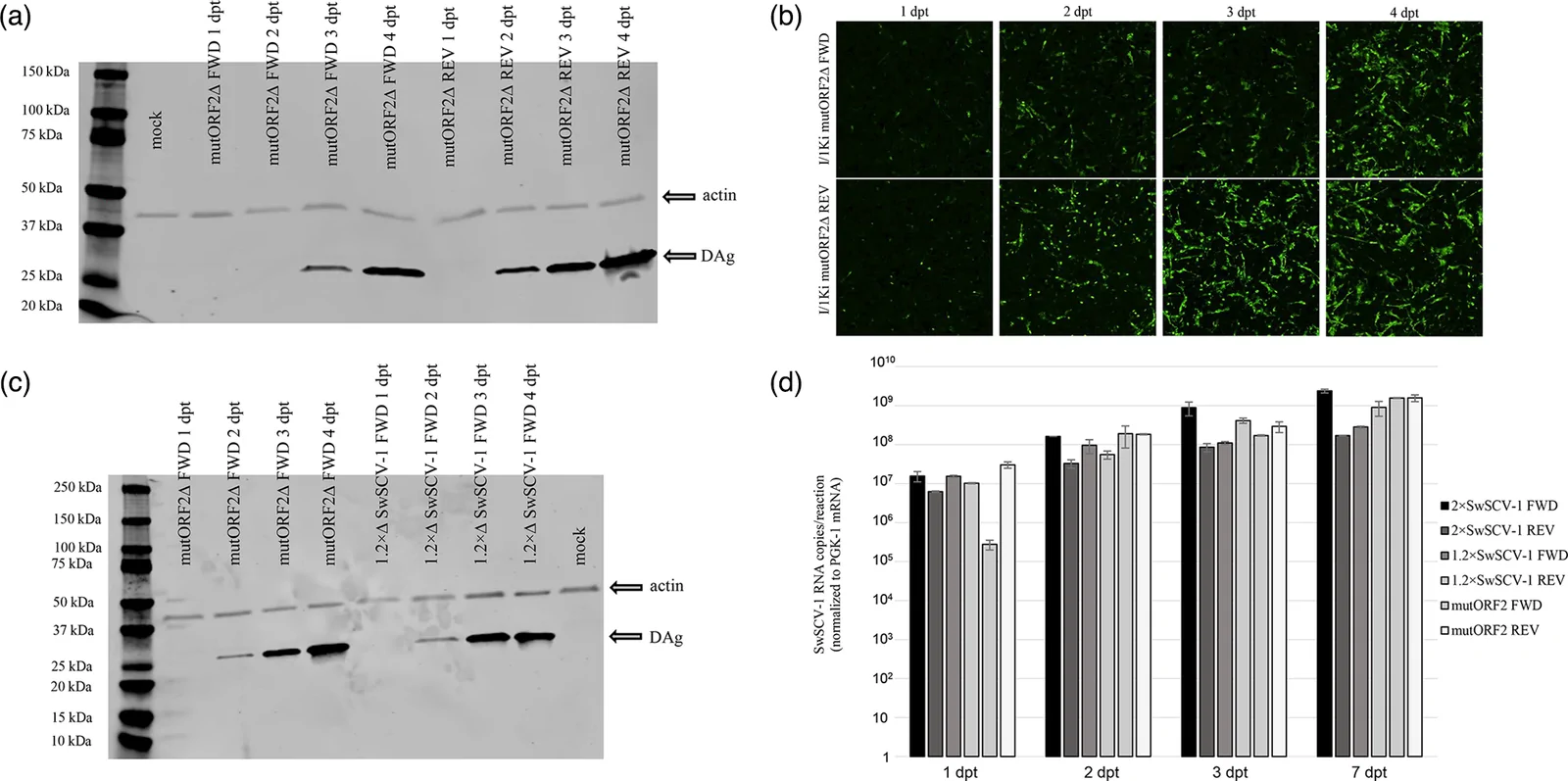
Transfection of mutORF2 SwSCV-1 constructs initiates replication in boa kidney cells. (**a**) I/1Ki cell pellets were collected 1–4 dpt with the mutORF2 SwSCV-1 FWD and REV constructs. The samples were separated on 4–20% Mini-PROTEAN TGX gels, transferred onto a nitrocellulose membrane, which was probed with rabbit α-SwSCV-1 DAg antiserum and mouse monoclonal anti-pan actin antibody. The results were recorded using the Odyssey Infrared Imaging System. (**b**) I/1Ki cells were fixed and stained for the DAg with rabbit α-SwSCV-1 DAg antiserum at 1–4 dpt with the mutORF2 SwSCV-1 FWD and REV constructs. AlexaFluor 488-labelled donkey anti-rabbit IgG served as the secondary antibody. The images were taken by the Opera Phenix High Content Screening System with a 10× objective. (**c**) WB comparison of DAg expression 1–4 days following transfection with the mutORF2 SwSCV-1 FWD and the previously described 1.2× genome SwSCV-1 FWD [27] constructs. The membranes were probed with rabbit α-SwSCV-1 DAg antiserum and mouse monoclonal anti-pan actin antibody and the results were recorded using the Odyssey Infrared Imaging System. (**d**) I/1Ki cells were transfected with 2×, 1.2× genome and mutORF2 SwSCV-1 FWD and REV constructs, followed by RNA isolation at 1–3 and 7 dpt. The samples were analysed by qRT-PCR after DNase I treatment, targeting genomic SwSCV-1 RNA. The results were normalized to PGK-1 mRNA. Each time point represents two biological replicates with two technical replicates. The y-axis shows SwSCV-1 RNA copy numbers/reaction and the error bars represent sd.

### ORF2 and its putative protein are not essential for persistent infection or infectious particle production

In our previous studies [17, 29], we observed that maintaining the cells after the initial transfection with the 2×SwSCV-1 FWD and 1.2×SwSCV-1 FWD constructs resulted in persistent infection of the cell line. To study if the ORF2 mutation would inhibit the establishment of persistent infection, we transfected I/1Ki cells with the mutORF2 SwSCV-1 FWD construct and passaged the cells to expand the culture, an approach earlier shown to result in persistent infection and loss of the original plasmid [17, 29]. IF and WB analyses of the cells revealed continued DAg expression, which, based on our earlier studies, we interpret to indicate persistent infection, implying that the putative ORF2 protein is not essential for replication. Roughly 1 year after the initial transfection, we compared the DAg expression and SwSCV-1 RNA levels in the three persistently SwSCV-1-infected cell lines (I/1Ki-2×Δ, I/1Ki-1.2×Δ, and I/1Ki-mutORF2Δ) by IF staining, Northern blot, qRT-PCR and WB (Fig. 3). IF staining of the I/1Ki-mutORF2Δ cells showed a similar level of infection in each of the cell lines (Fig. 3a). Investigation of RNA levels and DAg expression by Northern blot, qRT-PCR and WB did not reveal overt differences between the three persistently infected cell lines either (Fig. 3b–d). Even though the probe successfully detected *in vitro* transcribed control RNA, we were unable to detect DAg mRNA in the persistently infected cells, suggesting that the DAg mRNA level is below the detection limit.

**Fig. 3.**
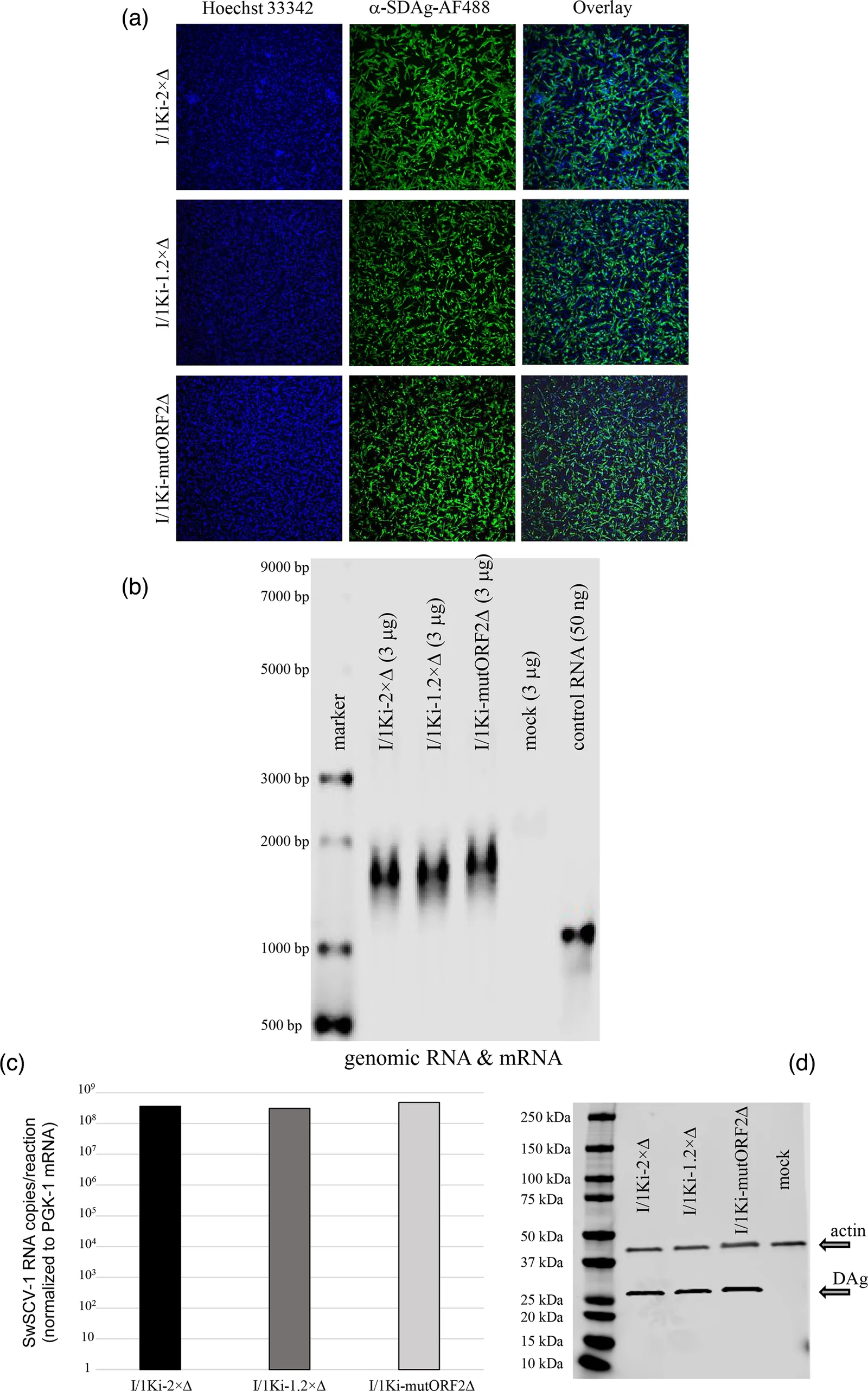
Comparison of the three persistently SwSCV-1-infected cell lines (I/1Ki-2×Δ, I/1Ki-1.2×Δ and I/1Ki-mutORF2Δ). (**a**) The persistently infected cell lines were fixed and stained using rabbit α-SwSCV-1 DAg antiserum and Alexa Fluor 488-labelled donkey anti-rabbit secondary antibody, while Hoechst 33342 served for staining the nuclei. The images were taken 3.5 years (I/1Ki-2×Δ), 14 months (I/1Ki-1.2×Δ) and 1.5 years (I/1Ki-mutORF2Δ) post-transfection using the Opera Phenix High Content Screening System with a 10× objective. (**b**) Total RNA was isolated from I/1Ki-2×Δ, I/1Ki-1.2×Δ, I/1Ki-mutORF2Δ and clean I/1Ki cells. An *in vitro* transcribed control RNA (~850 nts long) served as control. The RNA samples were separated on agarose-formaldehyde gel and transferred onto nylon membrane, which were probed for SwSCV-1 genomic RNA and SwSCV-1 DAg mRNA. The Odyssey Infrared Imaging System served for recording the results. (**c**) RNA was isolated from I/1Ki-2×Δ, I/1Ki-1.2×Δ and I/1Ki-mutORF2Δ cells and analysed by qRT-PCR targeting SwSCV-1 genomic RNA. The results were normalized to PGK-1 mRNA. The y-axis shows SwSCV-1 RNA copy numbers per reaction. (**d**) Samples of I/1Ki-2×Δ, I/1Ki-1.2×Δ, I/1Ki-mutORF2Δ and clean I/1Ki cells were separated on 4–20% Mini-PROTEAN TGX gels, transferred onto nitrocellulose membranes and probed with rabbit α-SwSCV-1 DAg antiserum and mouse monoclonal anti-pan actin antibody. The results were recorded using the Odyssey Infrared Imaging System.

To test if the mutation introduced in the ORF2 would interfere with infectious particle formation, we superinfected all three cell lines 3 weeks (thereby representing acute SwSCV-1 infection) after the initial transfection (2×, 1.2× and mutORF2 SwSCV-1 FWD) with HISV-1 (a hartmanivirus) a potent helper virus of SwSCV-1 [17]. Subsequently, we collected supernatants(SNTs) from the superinfected cells up to 9 days post-infection (dpi) and used them to inoculate naïve I/1Ki cells, which we analysed by IF staining at 4 dpi for DAg expression. The IF staining (Fig. 4a) of the cells inoculated with the SNTs collected from the superinfected cells at 3, 6 and 9 dpi showed similar staining patterns and similar levels of infectious particles. Quantification of the infectious SwSCV-1 particles formed after superinfection of the three cell lines confirmed the numbers of infectious particles secreted from all cell lines to be in the same range (Fig. 4b).

**Fig. 4.**
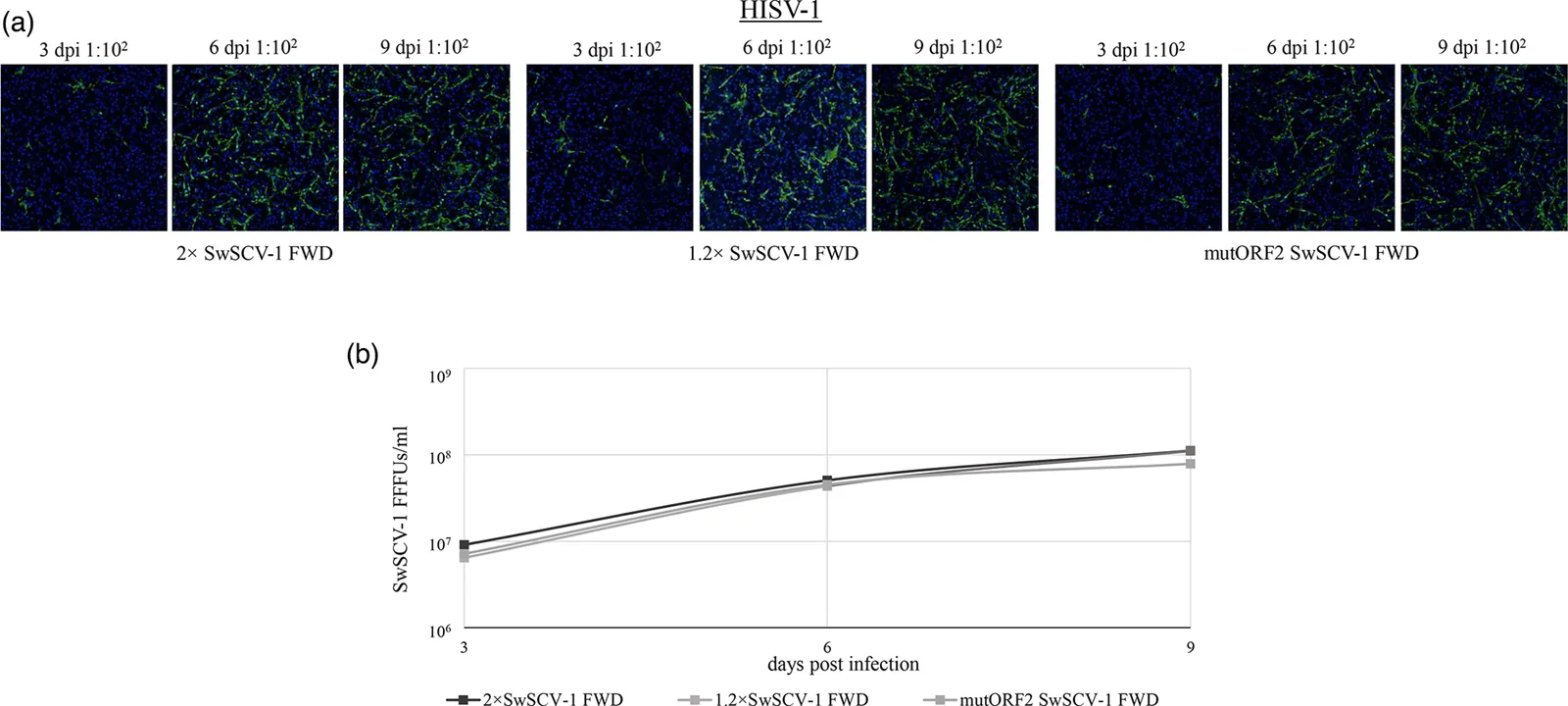
Comparison of the infectious particle production of the three freshly SwSCV-1-transfected (2×, 1.2× and mutORF2 SwSCV-1 FWD) cell lines. (**a**) The three freshly transfected I/1Ki cell lines were superinfected with HISV-1, supernatants were collected 3, 6 and 9 dpi and subsequently used for titration on naïve I/1Ki cells. Four days post-inoculation, we fixed and stained the cells using rabbit α-SwSCV-1 DAg antiserum and Alexa Fluor 488-labelled donkey anti-rabbit secondary antibody, while Hoechst 33342 served for staining the nuclei. The images were taken by the Opera Phenix High Content Screening System with a 10× objective. (**b**) The number of infected cells was counted using the Opera Phenix High Content Screening System, followed by quantification of infectious particles per millilitre of growth medium in terms of fluorescent f.f.u. values (displayed on y-axis).

### Passaging of SwSCV-1-infected cells leads to re-establishment of the ORF2

To study if persistent infection would induce mutations in the SwSCV-1 genome, we performed RNA sequencing of the three persistently SwSCV-1-infected cell lines (I/1Ki-2×Δ, I/1Ki-1.2×Δ and I/1Ki-mutORF2Δ) analysed >1 year after initiation of infection through transfection. The consensus sequences generated using Bowtie2 [44] from the RNA sequencing reads showed the presence of wild-type SwSCV-1 genome in the I/1Ki-1.2×Δ cells. However, the consensus sequence generated from the I/1Ki-2×Δ cells revealed a T to C point mutation at position 238 (GenBank accession no. MH988742.1) (TGGACTCCGGCGTTACTCGCATGGG T→C) in the SwSCV-1 genome. The same position showed high T to C variation also in the case of I/1Ki-1.2×Δ and I/1Ki-mutORF2Δ cell lines, and interestingly, the mutation would introduce a methionine codon in the antigenomic sense (CGTAT→CGCAT). The resulting ORF would only be ~15 amino acids long, thereby likely not representing a true protein. Curiously, the reference assembly of the SwSCV-1 genome from the I/1Ki-mutORF2Δ cell line showed that the introduced mutation had fully reverted during persistence (>1 year of infection), re-introducing the methionine initiation codon of the ORF2. Specifically, the position of the altered nt was represented by 620 reads, and the nt distribution at the mutated position was A=43 (6.9%), C=3 (0.5%), G=572 (92.3%) and T=2 (0,3%), indicating a strong preference for the nt present in the wild-type sequence. When we analysed the transfected cells by RT-PCR and Sanger sequencing at ~2 months post-initial transfection, we did not observe the reversion, likely due to its low abundance at that point.

### The putative protein product of ORF2 is cytoplasmic and SwSCV-1 infected snake does not have a detectable ORF2 antibody response

After observing reversion of the mutation in the ORF2 during persistent infection, we decided to investigate if we could detect the putative ORF2 protein product in SwSCV-1-infected I/1Ki cells. We attempted to generate rabbit antiserum against the His-tagged ORF2 protein product expressed in bacteria, as well as two selected peptides of the putative protein. Both approaches failed to produce an antiserum suitable for WB detection of the putative ORF2-encoded protein expressed in I/1Ki cells from constructs with and without a C-terminal HA-tag. Utilizing anti-HA antibody, we observed boiling and/or addition of reducing agent (DTT) to render the ORF2 protein undetectable in WB. After identifying a suitable sample pre-treatment, WB analysis of I/1Ki cell pellets collected 3 days post-ORF2-HA and ORF2 expression construct transfection, we found the putative protein to migrate at a rate approximately similar to the 20 kDa molecular weight marker (Fig. 5a). We hypothesized that if the protein is expressed at high levels during infection, an infected animal would produce antibodies against the ORF2 protein product. To test this hypothesis, we prepared a WB membrane of I/1Ki cells transfected with wild-type and mutORF2 SwSCV-1 (1.2×SwSCV-1 FWD and mutORF2 SwSCV-1 FWD) infectious clones, persistently SwSCV-1-infected (I/1Ki-1.2×Δ) I/1Ki cells and I/1Ki cells transfected with the ORF2 expression constructs (with or without HA-tag), and probed the membrane with the serum of the original SwSCV-1-infected boa constrictor [16]. The results showed that the snake serum indeed reacted with the DAg in all SwSCV-1-transfected/infected samples. However, it did not react with the ORF2 produced in I/1Ki through recombinant expression (Fig. 5a). The snake serum did not produce a similar sized band in any of the transfected/infected cell lysates either, suggesting that ORF2 would not be expressed during SwSCV-1 infection or that the putative protein would be poorly immunogenic and/or expressed at very low level.

**Fig. 5.**
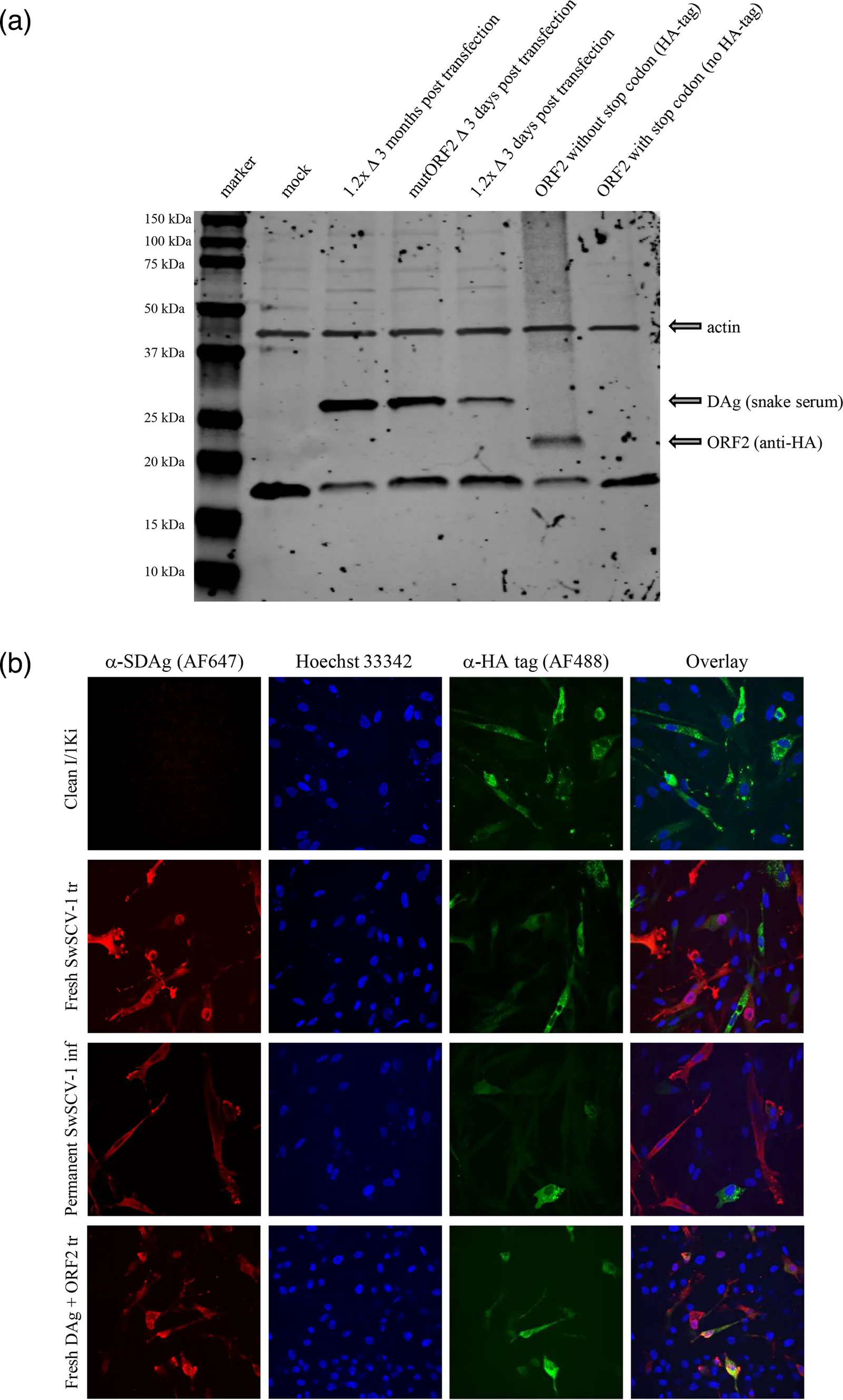
Comparing ORF2 expression patterns in freshly or persistently SwSCV-1-infected cells versus those in cells transfected with an ORF2 expression construct by WB and IF staining. (**a**) Samples of persistently SwSCV-1-infected, freshly SwSCV-1-transfected, ORF2 (with or without an HA-tag) expression construct transfected and clean I/1Ki cells were separated on 4–20% Mini-PROTEAN TGX gels and transferred onto nitrocellulose membranes. The membrane was probed with the serum of a SwSCV-1-infected snake at 1 : 250 dilution [16], anti-HA antibody at 1 : 1,000 dilution, and mouse monoclonal anti-pan actin antibody. The results were recorded using the Odyssey Infrared Imaging System. (**b**) Recombinant expression of the ORF2-HA protein in clean I/1Ki cells (top row), freshly SwSCV-1 transfected cells (second row), persistently SwSCV-1-infected cells (third row), and snake DAg-transfected cells (bottom row). The ORF2 protein shows cytoplasmic subcellular localization with a granular staining pattern and does not co-localize with the DAg. The cells were fixed and stained using rabbit α-SwSCV-1 DAg antiserum with Alexa Fluor 647-labelled donkey anti-rabbit secondary antibody, mouse α-HA primary antibody with Alexa Fluor 488-labelled donkey anti-mouse secondary antibody, while Hoechst 33342 served for staining the nuclei. The images were taken using the Opera Phenix High Content Screening System with a 63× objective.

To further study the putative protein product of ORF2, we transfected I/1Ki cells with expression constructs for HA-tag bearing ORF2 and used IF staining to study the subcellular localization of the protein in non-infected, SwSCV-1-infected or SwSCV-1 DAg-transfected I/1Ki cells. The IF staining showed a fairly intense granular or grainy staining pattern in the cytoplasm for the putative ORF2 protein in the uninfected cells (Fig. 5B). However, in cells infected with SwSCV-1 for <4 months (1.2×SwSCV-1 FWD), persistently (>4 months) SwSCV-1-infected (I/1Ki-1.2×Δ) cells or cells co-transfected with SwSCV-1 DAg, the ORF2 protein staining was considerably weaker. These observations could imply competition between the plasmid and the virus from the cellular RNA polymerase II’s resources or ORF2-DAg interaction mediated relocation, as ORF2 and DAg did not appear to colocalize (Fig. 5B).

### Directional RNAseq suggests that ORF2 is not transcribed to mRNA

To investigate the transcriptional patterns of SwSCV-1 in more detail, we performed strand-specific RNAseq on I/1Ki cells transfected with the 1.2×SwSCV-1 FWD infectious clone. The method preserves the polarity of the transcripts and allows us to determine the orientation of the reads and to identify the mRNA species within the cells. We utilized two approaches for selecting the RNA species for library preparation, poly(A) enrichment and ribosomal RNA (rRNA) depletion. Poly(A) enrichment focuses on mRNA species with polyadenylation, whereas rRNA depletion offers a more comprehensive representation of cellular RNA, including non-coding transcripts. We aligned the reads representing the original RNA template orientation to the 1.2×SwSCV-1 genome using Bowtie2 to allow the assessment of reads spanning the circular junctions of the genome. The genome is the most abundant viral RNA species during HDV replication [4], and indeed the mapping showed a strong bias towards the genomic strand in the case of reads obtained following rRNA depletion (Fig. 6A). The reads obtained from poly(A)-enriched RNA showed the presence of DAg mRNA (Fig. 6B) with a small bias towards the 5′ end of the mRNA. We did not locate a classical AAUAAA polyadenylation signal following the ORF2; however, there are both AAGAAA and GATAAA signals, these can theoretically be utilized for polyadenylation [45]. Neither the rRNA depletion nor poly(A) enrichment of the RNA prior to library preparation produced an obvious increase in reads covering the ORF2 (Fig. 6). In addition to DAg mRNA, we observed a high amount of reads mapping to the opposing side of the rod (Fig. 6B), perhaps mediated through co-purification of the opposing RNA strand (reflecting the self-complementary structure of kolmivirus) rather than direct polyadenylation-mediated enrichment of this region.

**Fig. 6.**
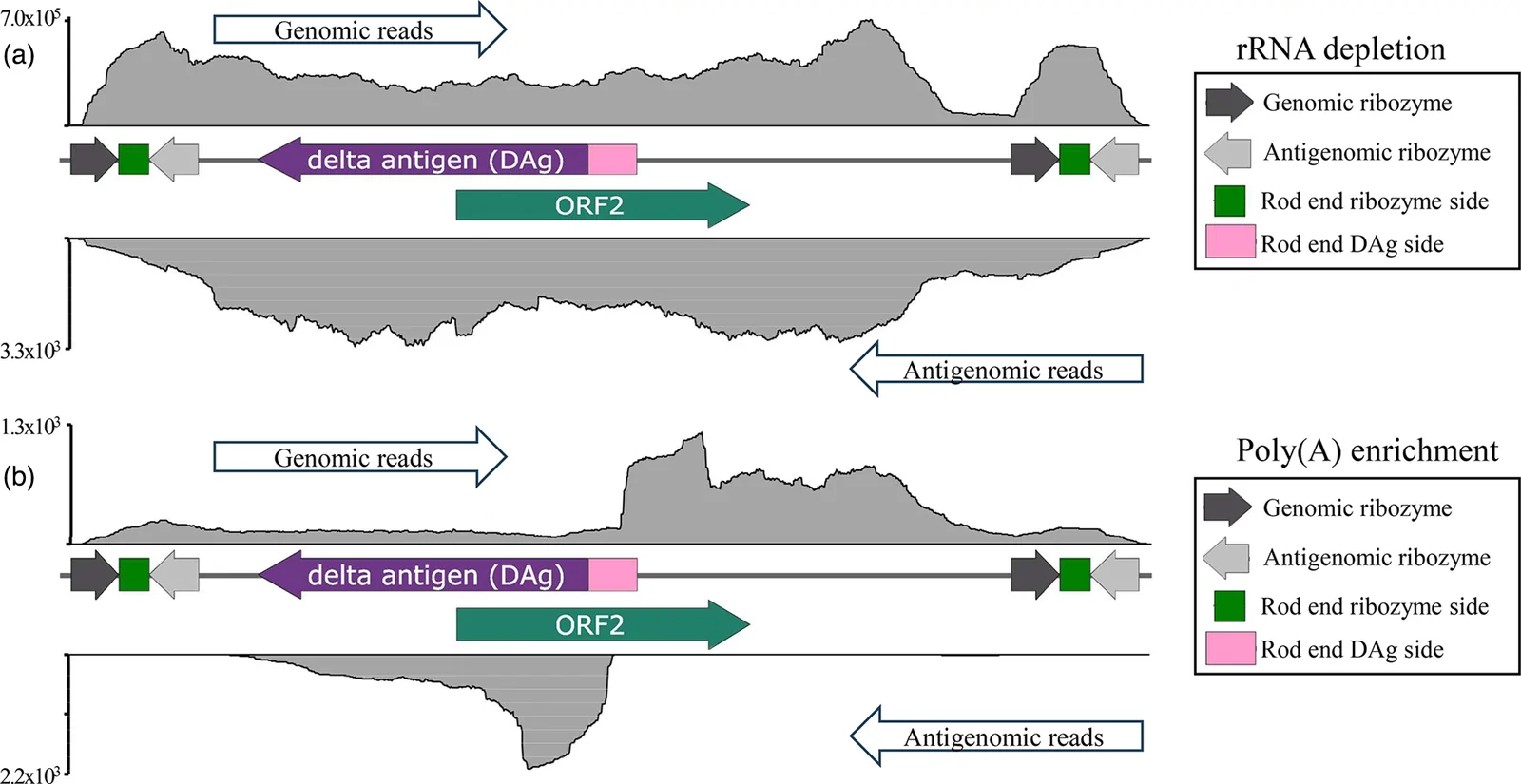
Directional RNAseq coverage maps of I/1Ki cells 4 weeks post-transfection with the 1.2×SwSCV-1 FWD infectious clone. Libraries were prepared using either rRNA depletion or poly(A) enrichment. The obtained reads were aligned to the 1.2×SwSCV-1 genome using Bowtie2. The y-axis shows the number of genomic and antigenomic mapped reads. (**a**) rRNA-depleted libraries show a strong bias towards the genomic strand, consistent with the genome being the most abundant RNA species during kolmiovirid replication. (**b**) Poly(A)-enriched libraries show reads corresponding to the DAg mRNA, while coverage over the ORF2 indicates low or absent mRNA transcription of the region.

### SwSCV-1 does not express the large form of the delta antigen

To further characterize the SwSCV-1 genome, we investigated whether its DAg has two forms. In the case of HDV, the two forms of DAg, S- and L-DAg, mediate different functions [12]. The HDV S-DAg prominently localizes to the nucleus and is critical for replication [46] whereas the L-DAg associates with particle formation [47]. In our earlier studies, when investigating infected tissues and cultured cells, we observed two (or more) bands in WBs when staining for SwSCV-1 DAg [16, 17], and speculated that the two bands could correspond to the S- and L-DAg. We generated three constructs for recombinant expression of the SwSCV-1 DAg, one with the unedited SwSCV-1 DAg ORF, the second with the S-DAg ORF only and the third with the DAg ORF in which the stop codon was replaced by a tryptophan codon, forcing L-DAg translation (Fig. 1C). We transfected I/1Ki cells with the constructs and analysed the protein expression by IF staining. This demonstrated similar if not identical staining patterns for the three DAg expression constructs (Fig. 7). We then compared the electrophoretic mobility of the DAg produced via plasmid expression to DAg produced by the persistently SwSCV-1-infected (Fig. 8A) and freshly SwSCV-1-transfected cell lines (Fig. 8B). The WB showed that both the freshly infected cells and the persistently infected cell lines only expressed the S-DAg form. This suggests that SwSCV-1 only expresses and requires a single DAg form for replication and infectious particle formation. To study whether the L-DAg expression occurs in cell lines of different tissue or host origin, we used the persistently SwSCV-1-infected snake cell lines described earlier (V/5Lu-Δ, V/1Ki-Δ, V/2Hz-Δ, and V/5Liv-Δ) [17] and a recently generated one (V/4Br-Δ), as well as two human cell lines (Huh7 and HEK293T) and compared their DAg expression by WB. The results show that regardless of the tissue or host origin of the cell line, there is only a single band, corresponding to the S-DAg (Fig. 8C–D).

**Fig. 7.**
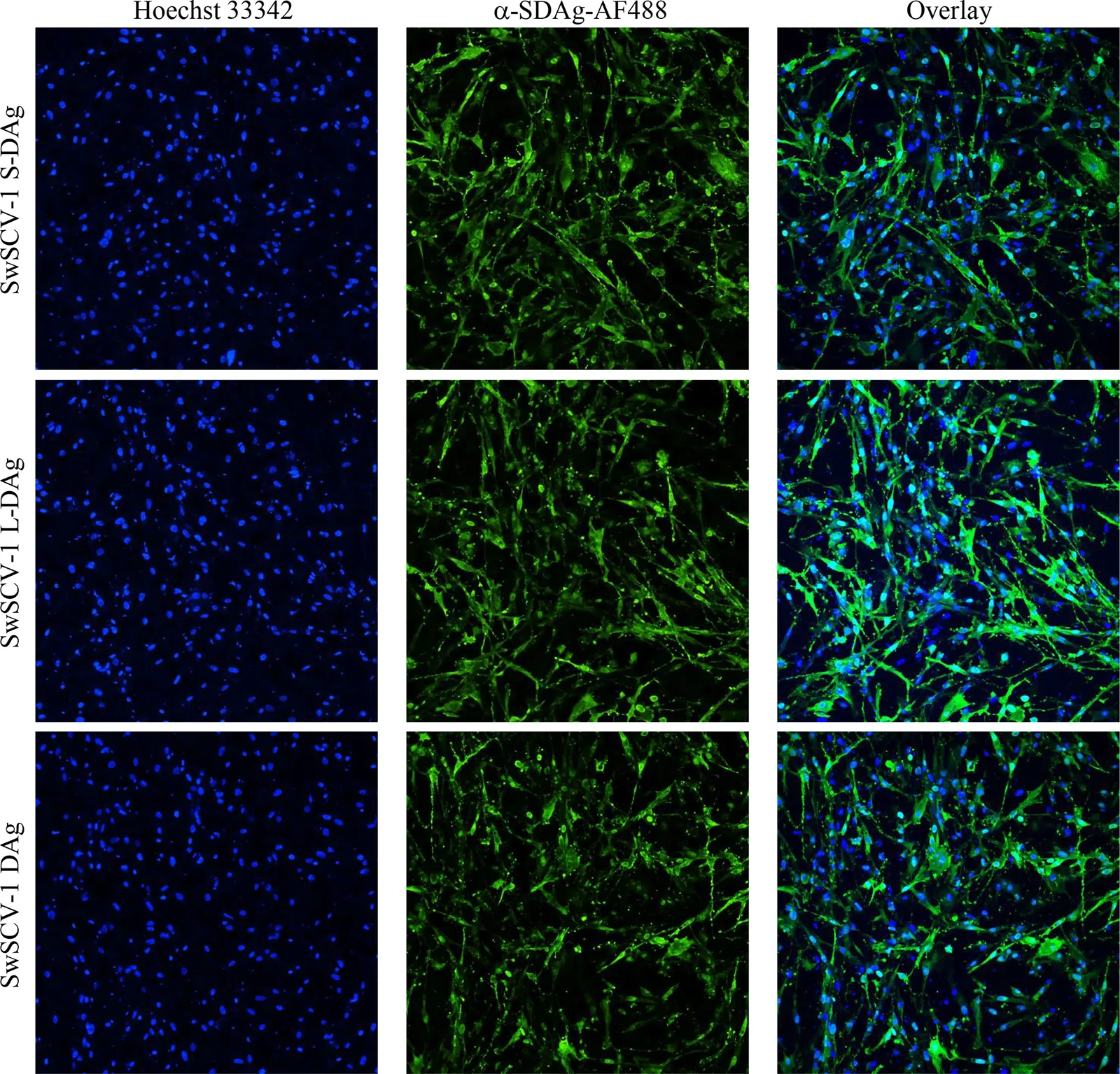
Comparison of protein expression after transfection with expression constructs. I/1Ki cells were transfected with plasmids bearing the small SwSCV-1 DAg (top panels), large SwSCV-1 DAg (second row panels) and the unedited SwSCV-1 DAg (third row panels). The cells were fixed and stained 3 dpt using rabbit α-SwSCV-1 DAg antiserum and Alexa Fluor 488-labelled donkey anti-rabbit secondary antibody, while Hoechst 33342 served for staining the nuclei. The images were taken using the Opera Phenix High Content Screening System with a 20× objective.

**Fig. 8.**
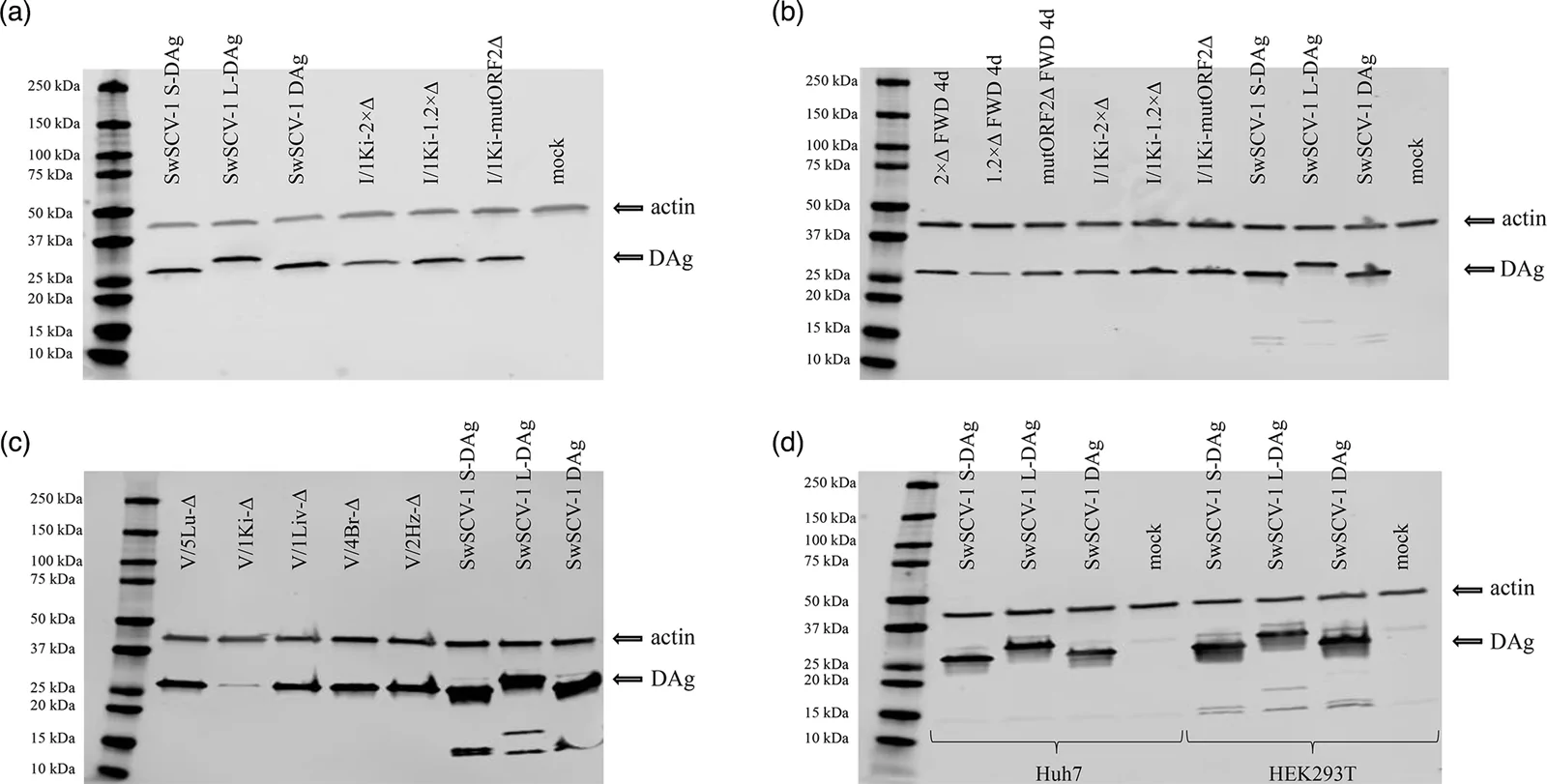
SwSCV-1 does not express large form of the DAg. (**a**) WB comparison of I/1Ki cells transfected with SwSCV-1 S-DAg, L-DAg and unedited DAg bearing construct and I/1Ki-2×Δ, I/1Ki-1.2×Δ and I/1Ki-mutORF2Δ cells. (**b**) WB comparison of freshly SwSCV-1-transfected cells, persistently SwSCV-1-infected cells and I/1Ki cells transfected with the SwSCV-1 S-DAg, L-DAg and DAg expression constructs. (**c**) WB comparison of persistently SwSCV-1-infected cell lines originating from various tissues of boa constrictor snakes and cells transfected with the SwSCV-1 S-DAg, L-DAg and DAg expression constructs. (d) WB comparison of human hepatocellular carcinoma (Huh7) and human embryonic kidney (HEK293T) cells transfected with the SwSCV-1 S-DAg, L-DAg and DAg expression constructs. Cell pellets were collected 3 dpt. (**a–d**) The proteins were separated on 4–20% Mini-PROTEAN TGX gels, followed by transfer onto nitrocellulose membranes. The membranes were probed with rabbit α-SwSCV-1 DAg antiserum and mouse monoclonal anti-pan actin antibody.

## Discussion

Owing to almost half a century of research since the discovery of HDV [1], a wealth of knowledge on the unique features of HDV is available. However, the newly identified distinct HDV-*like* agents from a wide range of taxa [15, 16, 18–21], classified in different species within the family *Kolmioviridae* of the realm *Ribozyviria* [25, 26], have brought new dimensions to the research around these enigmatic agents. So far, however, reports have characterized only some of the novel agents further than genome description, calling for studies comparing them against the best-known member, HDV. After identifying SwSCV-1, we have been developing molecular biology tools that allow us to study SwSCV-1 more extensively. So far, we have shown its ability to initiate replication *in vitro* following the transfection of a dimer or 1.2× genome constructs [17, 29], in a similar manner to HDV [48–50]. In the present study, we focused on the ORFs present in the SwSCV-1 genome. For HDV, the early studies showed that during replication only the DAg ORF appears to be translated [5]. A recent interesting study indicated that an additional protein would be translated from the *S. canaria*-associated deltavirus genome [28], further emphasizing the importance of investigating the novel kolmiovirids in more depth. The genome of SwSCV-1 harbours two partially overlapping >500 base ORFs, the DAg ORF in the antigenomic orientation and the ORF2 in the genomic orientation (Fig. 1A). The putative protein encoded by ORF2 does not show substantial similarity to any known protein. Since the sequence of the putative *S. canaria*-associated deltavirus ORF2 is not available, we were unable to perform a comparison. Therefore, we investigated whether disruption of ORF2 affects the replication capacity of SwSCV-1. In addition, we examined whether the SwSCV-1 DAg is expressed in two distinct forms, as reported for HDV [7].

To explore the possible roles of the putative ORF2 protein, we generated a construct in which an isoleucine codon replaces the ‘wild-type’ methionine initiation codon of the SwSCV-1 ORF2. By comparing the ‘mutORF2’ construct to wild-type SwSCV-1 genome-bearing constructs, we showed that eliminating the possibility of protein translation does not inhibit initiation of replication or the ability to establish persistent infection (Figs. 2 and 3). Our results also showed that the introduced mutation did not markedly affect the ability of infectious SwSCV-1 particle formation upon superinfection with a potent helper virus (Fig. 4).

RNA sequencing of the persistently SwSCV-1-infected cells did not reveal significant changes in the genome of the virus. We identified a single nt mutation in the consensus sequence of SwSCV-1 obtained from cells transfected with the 2×SwSCV-1 FWD construct ~3.5 years earlier. The other persistently infected cell lines showed variation at the same sequence position, but the altered nt had not yet become dominant in those cell lines. However, both the wild-type and the mutated sequence appeared abundant in the genome of SwSCV-1 obtained from all the persistently infected cell lines. It seems unlikely that the single nt mutation outside the DAg ORF and the ORF2 would dramatically affect the viral life cycle. On the other hand, the point mutation could alter the pairing between the opposite sides of the circular ssRNA genome, thereby affecting the transcription. The sequencing further revealed that the mutation introduced into the ORF2 initiation codon had reverted in the persistently infected cells. The reversion was not present in the freshly transfected cells analysed 2 months after transfection by Sanger sequencing. We speculated that the introduced nt change ‘de-optimized’ the respective amino acid codon in the DAg ORF as the nt change from AGCAT to AGTAT changes a serine codon of the DAg ORF from AGC to AGT. However, investigation of the serine codons within the DAg showed the AGT codon to be more abundant. Furthermore, both of these codons are approximately equally abundant in the case of *Python molurus bivitattus* (codon usage table for boa constrictor not available) (Codon Usage Database, https://www.kazusa.or.jp/codon/). Together these data suggest that codon usage bias would be an unlikely explanation for the mutation’s reversion. An alternative explanation could be that the mutation alters the secondary structure of the genomic or antigenomic RNA, and reversion of the mutation restores the virus’ replication kinetics. In the end, however, we cannot rule out the possibility that the ORF2 itself would be beneficial for the virus at some stage of the replication cycle, even if we could not find evidence for expression of the protein product.

By transfecting ORF2 expressing constructs into boa kidney cells, we were able to confirm that a stable protein can be translated from the ORF. Although we were unsuccessful in our trials to generate an ORF2-specific antibody, the addition of an HA-tag into the protein expressing constructs allowed subsequent detection of ORF2 both in IF staining and WB. Co-expression of the putative protein and DAg did not alter the staining pattern of either protein nor did they co-localize, suggesting that the proteins would be unlikely to interact. The serum of the snake in which we originally identified SwSCV-1 [16] failed to detect the putative ORF2-encoded protein, suggesting that the protein is not expressed during the viral life cycle or that no antibodies are produced against it due to, e.g. low immunogenicity, very low expression levels or a complete lack of its expression.

Directional RNA sequencing of cells transfected with 1.2×SwSCV-1 FWD and analysed at 4 weeks post-transfection indicated a transcript from the DAg ORF as expected. During poly(A) selection in library preparation, mRNAs are captured by their 3′ end poly(A) tail, causing a possible bias in the amount of reads towards the 3′ end of the transcript, which we could observe in the case of the DAg. In contrast, the sequencing data could not convincingly demonstrate the presence of ORF2 mRNA (Fig. 6B); however, it does not completely rule out its possible presence. The kolmiovirus genomes form rod-*like* structures through extensive internal base pairing of the circular single-stranded genome, and the ORF2 spans the ‘rod-end’ of the genome. Considering the fact that the ‘rod-end’ of the genome end acts as the transcriptional promoter of HDV [51, 52], it would seem unlikely that an actively employed ORF would span this region. The ORF2 lacks a downstream conventional poly(A) signal; however, there are two non-conventional poly(A) signals present downstream of ORF2 which could imply that the protein product of this ORF can be translated in some cells or tissues. Interestingly, poly(A) selection revealed reads mapping to the SwSCV-1 rod on the opposing side of the DAg ORF (Fig. 6B). This could indicate that similarly to HDV [52], the rod end functions as a bidirectional promoter. These observations would merit further research.

In the case of HDV, the S- and L-DAg carry different roles during the viral life cycle [7, 8, 12]. In our earlier studies, using WB, we have detected double bands when staining for SwSCV-1 DAg, leading us to speculate the two bands to putatively correspond to the S- and L-DAg. However, here, using recombinant proteins, we could conclude that the persistently SwSCV-1 infected I/1Ki cells mostly, if not solely, produce S-DAg (Fig. 8A), and the double bands observed in earlier studies are likely the result of post-translation modification of the DAg or proteolytic degradation. This would be in line with the findings that some other non-HDV kolmioviruses only express a single form of the DAg [21, 53, 54]. In addition, Wille and co-authors did not, based on sequence analysis, find a putative ADAR editing site in the DAg of dabbling duck virus 1 [15]. Iwamoto and colleagues found no possible editing site in the sequences of *Taeniopygia guttata* and *Marmota monax* deltaviruses by looking at nt variation, while they identified a potential RNA-editing site in the sequence of *Odocoileus virginianus* deltavirus; however, the elongation would be only with two amino acids [19]. Tome’s spiny rat virus likewise contains an amber stop codon, suggesting the potential for ADAR-mediated editing [54]. However, neither knocking out nor overexpressing of the ADAR altered the expression pattern of the rodent DAg as assessed by WB analysis [54]. Because we had in our earlier studies observed double or multiple bands for DAg in WB of some samples [16, 17, 29], we further studied the possibility that the ADAR editing resulting in L-DAg translation would occur in cell lines originating from different tissues. Further WB analysis of both freshly and persistently SwSCV-1-infected I/1Ki cells, different snake cell lines infected with SwSCV-1 and of human cell lines transfected with the SwSCV-1 DAg expression constructs all showed only the presence of S-DAg (Fig. 8B–C), suggesting that the expression levels or lack of ADAR expression in different cell types would not affect the S- versus L-DAg in the case of SwSCV-1. We currently lack the tools to confirm ADAR expression or to assess its expression levels in boa constrictor-derived cell lines. Moreover, RNA sequencing data from the I/1Ki cells indicate that only a negligible number of reads showed variation at the S-DAg stop codon. The findings would indicate that ADAR editing does not occur in our boa constrictor cell lines under the conditions tested. Furthermore, they may imply that ADAR editing responsible for the elongation of DAg would specifically apply to HDV, and it could relate to co-evolution with HBV in humans.

Although absence of evidence is not evidence of absence, we think that our results provide strong evidence that ORF2 is not translated during infection. Specifically, the experiments with the mutORF2 construct showed that removal of the methionine codon does not markedly affect replication, establishment of persistent infection or particle formation. However, the methionine codon reappeared during persistent infection, suggesting the original ORF2 sequence to be beneficial for the virus. The nt altered changed serine codon in the DAg ORF, and reversion to the serine ‘wild-type’ codon might explain the observed results. Whether similar reversion would occur in some other DAg codons might provide additional evidence to rule in or out ORF2 protein expression. Although we did not find any other evidence supporting ORF2 protein expression, we cannot fully eliminate the possibility that the protein might be expressed during very specific periods of the replication cycle and/or in very small amounts, thus remaining under the detection threshold. However, the absence of antibodies against the putative protein in SwSCV-1-infected animals and the data from the strand-specific RNA sequencing are additional evidence agreeing with the interpretation that no protein is being translated from the ORF2. Even though SwSCV-1 seems to express a single protein, similarly to HDV, the translation of additional ORFs in the *Kolmioviridae* family warrants further studies. Such studies could be aided by generation of sensitive detection tools for the proteins encoded in the additional ORFs. It is possible that not all ORFs would require polyadenylation for protein expression, e.g. hantavirus and arenavirus mRNAs are not polyadenylated. By reporting our findings, we hope to highlight the importance of studying the potential of additional ORFs in kolmiovirids, which seems to be a surprisingly overlooked topic in the currently existing literature.

## Abbreviations

ADAR: adenosine deaminase acting on RNA
DAg: delta antigen
dpi: days post-infection
dpt: days post-transfection
FWD: forward
HBV: hepatitis B virus
HDV: hepatitis D virus
HISV-1: Haartman Institute snake virus 1
IF: Immunofluorescence
LB: Luria broth
L-DAg: large delta antigen
NGS: next-generation sequencing
O/N: overnight
PFA: paraformaldehyde
PGK-1: phosphoglycerate kinase 1
qRT-PCR: quantitative reverse transcription polymerase chain reaction
rRNA: ribosomal RNA
RT: room temperature
S-DAg: small delta antigen
SNT: supernatant
SwSCV-1: Swiss snake colony virus 1
WB: Western blot
